# Influence of behavioral determinants on deviation of body mass index among 12-15 years old school children of Panchkula

**DOI:** 10.4178/epih/e2014021

**Published:** 2014-09-29

**Authors:** Amandeep Chopra, Nanak Chand Rao, Nidhi Gupta, Shelja Vashisth, Manav Lakhanpal

**Affiliations:** Department of Public Health Dentistry, Swami Devi Dyal Dental College, Panchkula, Haryana, India

**Keywords:** Body mass index, Diet, Socioeconomic status

## Abstract

**OBJECTIVES:**

To evaluate the body mass index (BMI) and factors related to BMI in 12-15 years old adolescents attending school in the Panchkula district of Haryana, India.

**METHODS:**

Our multistage sampling method enrolled 810 adolescents. Demographic data and dietary history data over 5 days were recorded. Height and weight were measured to calculate BMI, which was further categorized according to the World Health Organization classification system. Diet was analysed using the Nizel criteria and socioeconomic status (SES) was assessed using Prasad’s socioeconomic classification. The chi-squared test and analysis of variance test were performed, and a multinomial regression analysis was performed to find significant correlates with BMI.

**RESULTS:**

The prevalences of underweight, normal weight, overweight, and obesity were 13.6, 58.4, 22.7, and 5.3%, respectively. The prevalence of both overweight and obesity was higher among males than that among females. The overall food group, nutrient, sweet, and oral health diet scores were higher among overweight and obese adolescents. Adolescents attending public school were 2.62 times more likely than private school adolescents were to be underweight. Private school adolescents were 2.08 times more likely than public school adolescents were to be overweight. Those with a high SES, vegetarians, and those aged 15 years were highly likely to be obese.

**CONCLUSIONS:**

We found 41.6% of these adolescents to have a BMI that deviated from the norm. Important factors related with BMI were age, gender, socioeconomic score, mean daily diet score, and the type of school.

## INTRODUCTION

Food and water are essential elements for human survival. Therefore, access to adequate, sufficient, and healthy nutrition is considered an important fundamental right and plays an important role in the developmental process. However, social inequalities, changes in lifestyle, industrialization, and other factors have negatively influenced the spread of this fundamental right [1]. Today, two of the biggest problems the world faces are hunger or nutritional deficiency and dietary excess. Urbanization and economic development has resulted in rapid changes in diet and lifestyles [2].

Body mass index (BMI) is considered a simple method to analyse a population’s nutritional status [3]. In the US, the prevalence of obesity has been reported as 39% [4]. However, in developing countries, malnutrition can be an important public health problem: the World Bank reported that India, for example, has the second largest population of children (47%) suffering from malnutrition in the world, after Bangladesh, where 48% of Bangladeshi children exhibit a degree of malnutrition [5]. This extremely high prevalence of underweight children in India is nearly double that of sub-Saharan Africa with dire risks of high morbidity and mortality as well as low productivity and economic growth. In other areas of the world, a small but increasing percentage of overweight children are at a high risk of developing non-communicable diseases such as diabetes and cardiovascular disease later in life [5]. Follow-up studies from childhood to adulthood have shown that overweight children may become overweight adults if obesity persists in the teenage years [6].

Therefore, we aimed to investigate whether BMI levels deviated among adolescents attending school in the Panchkula district of Haryana, India as well as factors related to BMI.

## MATERIALS AND METHODS

A six-month cross-sectional study was conducted from April to September 2013 on 12-15-year-old adolescents attending school in the Panchkula district of Haryana, India. Ethical clearance was obtained from our institutional ethics committee, and oral informed consent was obtained from all study participants. A pilot study was conducted one month prior to the original study with a sample of 80 adolescents who were not included in the main sample. Approximately half of the pilot study population had a BMI that deviated from the normal range. Based on these results, our minimum sample size was calculated to be 810 [7] (Appendix 1).

The Panchkula district was formed on August 15, 1995 as the 17th district in the state of Haryana, India (Appendix 2). According to the 2011 census, the population of Panchkula was 5,58,890 with a literacy rate of 83.4%, which is higher than that in Haryana and India (76.64 and 74.04%, respectively), and the population density is 622 per km^2^, which is greater than that in Haryana and India (577 and 382 per km^2^, respectively). A multistage sampling technique was adopted to select adolescents attending schools within four blocks in the district, namely in Barwala, Pinjore, Raipur Rani and Morni (Appendix 2). Schools in each block were randomly selected based on the number of private and public schools and the total number of attendees at each school. Before examining each participant, informed consent was obtained from administrators of each educational department and principal of each school in Panchkula. Any adolescent between the ages of 12-15 years who were available on the day of examination in the selected schools were examined until the desired sample size was achieved. Adolescents were excluded if they were found to have a chronic illness, severe malnutrition, endocrine problems, physical or mental defects, or apparent obesity induced by or associated with any syndrome. In addition, any adolescent who reported smoking or chewing tobacco during the previous 6 months or chose not to cooperate during anthropometric measurements were excluded.

One examiner was trained prior to the commencement of the study. In addition, a pre-survey calibration was performed on a group of 30 participants aged between 12-15 years who were chosen from the school oral health programme. These preliminary results were subjected to kappa statistics. The calibration exercise and the kappa value (0.95) were in good agreement for the intra-examiner variability; thus, the examination procedure was validated.

Participants were asked to fill out their demographic details, family income, and any relevant medical history and diet history over 5 days prior to the examination, and then after 5 days return it to the examiner.

To calculate BMI, the height and weight of each participant was recorded. Weight was measured to the nearest 0.1 kg while not wearing shoes using a portable glass electronic scale (EB829; Lakhani Medicare Pvt. Ltd., Faridabad, India). Height was measured to the nearest 0.5 cm using a portable height-measuring unit (Biocon™; size 200 cm, Bharat Enterprises, New Delhi, India). BMI was calculated by dividing participants’ weight in kilograms by their height in meters squared (kg/m^2^). Then, BMI data were categorized according to the World Health Organization classification for BMI as underweight (<18.50 kg/m^2^), normal (18.50-24.99 kg/m^2^), overweight (25-29.99 kg/m^2^) and obese (≥30 kg/m^2^) [8].

Diet analysis was done using the criteria published by Nizel [9]. Food items were classified based on the (i) food group (i.e., fruits and vegetables, breads and cereals, milk and dairy, and meats); (ii) list of nutrients, and (iii) type of sugar present. Based on this information, the food group score, nutrient score and sweet score were calculated. The total diet score was calculated by adding the food score and nutrient score, and then subtracting the sweet score.

Socioeconomic status (SES) was assessed using an updated version of Prasad’s socioeconomic classification [10]. In this calculation, the per capita monthly income was calculated from information provided by the parent or guardian. Based on this per capita monthly income, the adolescents were categorized into different SES groups as follows:

1. Upper high: a per capita monthly income of Indian rupee (Rs) 5,156 or above
2. High: a per capita monthly income of Rs 2,578-5,155
3. Upper middle: a per capita monthly income of Rs 1,547-2,577
4. Lower middle: a per capita monthly income of Rs 773-1,546
5. Poor: a per capita monthly income below Rs 773

All data were processed and analysed using IBM SPSS version 20.0 (IBM Corp., Armonk, NY, USA), Descriptive analyses were carried out to assess differences in BMI. Continuous measurements were presented as mean ± standard deviation (95% confidence interval), and categorical measurements were presented as number (percent). The chi-squared test and analysis of variance were used to evaluate significant differences across the categories of BMI with gender, age, SES, and other covariates. The level of statistical significance was set at p<0.05. In addition, multinomial regression analyses were performed to find variables significantly correlated with underweight and overweight/obese adolescents.

## RESULTS

This study population consisted of 810 adolescents, of which 459 (56.7%) were males and 351 (43.3%) were females. Among those aged 12 years, 13 years, 14 years and 15 years, 283 (146 males, 137 females), 193 (121 males, 72 females), 170 (105 males, 65 females) and 164 (87 males, 77 females) adolescents were surveyed, respectively (Table 1).

The prevalences of underweight, normal weight, overweight and obesity were 13.6, 58.4, 22.7, and 5.3%, respectively.

Table 2 gives distribution of BMI by gender, age, SES, school type, and diet. In the total population, 473 had a normal BMI, of which 251 (54.7%) were males and 222 (63.2%) were females. More males were underweight (16.1%) and obese (8.5%), whereas more females were overweight (25.4%). By age most of the underweight adolescents were 12 years (27.3%) and 13 years (30%) old. However, 35.5% of those 12 years old had a normal BMI, and 38% and 24.5% of those aged 12 and 13 years old were overweight, respectively. The majority of obese adolescents (37.2%) were 15 years old. When we studied the population based on SES most of the underweight adolescents belonged to the upper and lower middle income levels. The high and upper middle SES level had the majority of the overweight and obese adolescents. However, the high, upper, and lower middle SES categories had a large number of those with a normal BMI. Those attending private schools comprised 10.4%, 52.2%, 31.4%, and 6% of the underweight, normal weight, overweight and obese categories, respectively. However, those attending public government schools comprised 15.6%, 62.3%, 17.2% and 4.9% of the underweight, normal weight, overweight and obese categories, respectively. These values were found to be statistically significant (p<0.05). Based on type of diet vegetarians were 13.5% underweight, 57.6% normal weight, 22.9% overweight and 6% obese. However, among those eating a mixed diet, 14.3% were underweight, 63.4% were normal weight, 21.4% were overweight and 0.9% were obese.

Table 3 shows the mean daily diet score according to BMI. The overall food group, nutrient, sweet and oral health diet scores were 24.67, 40.86, 10.34 and 55 among the underweight adolescents; 27.15, 44.64, 10.45, and 60.57 among the normal weight adolescents; 29.81, 47.48, 15.01 and 62.29 among the overweight adolescents, and 29.88, 45.3, 15.76 and 58.41 among the obese adolescents, respectively.

Table 4 shows the results of the multinomial regression on the relationship between BMI and other covariates. Females were 1.72 times more likely than males to be underweight, whereas males were 7.5 times more likely than females to be overweight. Those who attended public schools were 2.62 times more likely than were those who attended private school to be underweight. However, private school adolescents were 2.08 times more likely than public school adolescents to be overweight. Among those with a high SES, vegetarians and those aged 15 years were at high likelihood of being obese.

## DISCUSSION

In developing countries, like India, lifestyle transitions and economic growth have contributed to decreased physical activity and altered eating patterns [11]. As nations industrialize and populations urbanize, the traditional plant-based diets are replaced by diets with more animal products and a higher proportion of added sugars and fats [12]. Modernization brings with it the ill effects of reduced outdoor activity, while encouraging snacking and an unprecedented abundance of cheap, energy-dense foods [13,14]. In the developing world, these nutritional transitions, which involve a prevalence of junk food and decreased physical activity, have been associated with rapidly growing rates of overweight and obesity [12]. Despite the escalating problem of obesity, being underweight also remains a major problem in developing countries [15]. Underweight can be due to malnutrition, which results from an unavailability of adequate food, insufficient intake of food or of certain nutrients, and inability of the body to absorb and use nutrients.

This cross-sectional study was simple and low cost, and data could be collected relatively quickly. Adolescents of both genders were included to take into account the gender differences for body fat due to differences in their growth milestones as well as body structure and hormonal effects. In addition, we included adolescents from both public and private schools to increase the diversity of the social, economic, and cultural backgrounds of this study population.

Recent studies have reported the prevalence of obesity among school children and adolescents in various parts of India to range from 3 to 29% [16]. In our study, the percentage of overweight and obesity was 22.7 and 5.3%, respectively. Our observed prevalence was higher than that reported by Thippeswamy et al. [17], but less than that reported by Chakravathy et al. [18]. These differences were likely due to discrepancies between the definitions of overweight and obesity, age groups and genders selected for study, uniformity of the study sample, region in India, and methodology employed.

The males were 7.5 times more likely than the females were to be overweight. These results are in concordance with those of previous studies conducted at Punjab [19] and Delhi, India [20] where the prevalence of overweight and obesity was higher among males than that among females. One of the principal predictors of BMI is the SES of the individual [21]. In the present study, we observed that 32.8%, 26.9% and 26% of these adolescents belonged to upper middle, lower middle and high SES, respectively. This distribution suggests that our participants came from a range of SES because they were selected from both affluent and non-affluent schools. However, the information provided by the participants’ parents may not be a true indicator of the actual parental income.

The relationship between overweight and SES varies across countries. Children from a high SES were more likely to be obese in India, China and Russia [22]. However, in the US, a low SES among children is associated with being overweight [23]. In contrast, a high percentage of underweight adolescents (37.3%) in our study were from a low SES background. However, a higher percentage of overweight (52.1%) and obese (48.8%) adolescents were from an upper high and high SES background. Furthermore, our regression analysis indicated that those from a high SES were more likely than those from a poor SES were to be overweight. Similarly, other studies have reported a significant increase in prevalence of overweight, affluent children [11, 24]. One reason might be that, in addition to BMI, SES also influences a child’s dietary intake since SES reflects a family’s ability to purchase foods, and more nutritious food items have been shown to be more expensive than less nutritious food items are [25].

In our study, adolescents from the lower middle SES consumed significantly less of all food groups as well as snacks and had a higher number of underweight adolescents than those in other SES groups did. However, evidence from several developed countries on SES and diet have found that those with a higher SES tend to have a healthier diet, characterized by a greater consumption of fruits, vegetables, and low-fat milk and with a lesser consumption of fats overall [25]. Thus, US children from low SES were found to consume more of the less expensive junk foods and are prone to be overweight [26].

In developing countries, several important changes have occurred with improvements in SES. These changes relate to an increased ability purchase foods and flexibility in the choice of diet and activities. The nutritional transition in India has shown a substitution of coarse grains by the highly polished cereals such as rice. In addition, a progressive increase in the intake of edible fats, sugars, and sweets has been noted [27]. In the present study, the mean daily intake of all food groups and snacks was observed to be higher among those of high SES.

BMI is influenced by daily dietary intake. A proper diet should consist of an adequate representation of all food groups. In our study, underweight adolescents showed a significantly lower food group score, nutrient score, sweet score, and oral health diet score than adolescents of other BMI level did. These lower scores may be a major contributing factor to their lower BMI. However, overweight and obese adolescent were observed to have a significantly higher food group score, nutrient score, sweet score, and oral health diet score. As fast food and eating outside the home becomes more prevalent, the frequency of snacking as well as the contribution of snacks to one’s total caloric intake will likely increase. Foods from fast food restaurants tend to be huge portions and of low nutritional value since fruits, vegetables, and dairy products are typically not available. Similarly, in our study, adolescents who consumed significantly more sugar were found to be overweight. This finding is in accordance with that of previous studies, which have shown an association between increased carbohydrate consumption, BMI, and obesity [27-29].

Results of our regression analysis revealed that those eating a vegetarian diet were more likely to be obese than those who ate a mixed diet were; however, previous studies have reported opposite findings [30]. This discrepancy may be because the majority of vegetarians in our study were also from a high SES.

The role of physical activity in body weight regulation remains ambiguous [28]. It has been suggested that children who are engaged in mostly sedentary activities may have different patterns of food consumption and snacking, thus affecting their BMI. Further investigation on the relationship between physical activity, gender, age, SES, and BMI are needed.

This study has several limitations. Firstly, since this was a cross-sectional study, causal relationships cannot be established and the observed association may be due to other unexplored factors. Secondly, only adolescents who attended school during the examination were studied. Finally, we only performed qualitative, not quantitative, analyses of the adolescents’ diets. In India, most diets are consumed in the home; therefore, it is difficult to assess the caloric value of these diets, which are important to understand the diet’s influence on BMI. In addition, risk factors for obesity could not be directly studied because this information was not available.

However, the main strength of our study is that it is the first study, to our knowledge, to provide an overview of the burden of obesity among adolescents aged between 12-15 years in Panchkula, Haryana, India. Moreover, public health personnel and decision makers may use our study for future comparisons.

In conclusion, the prevalences of underweight, normal weight, overweight and obesity were 13.6, 58.4, 22.75 and 5.3%, respectively. Important factors related with differences in BMI were age, gender, socioeconomic score, mean daily diet score, and whether they attended a public or private school. This study suggests that there is a need for structured school health programmes to create awareness of weight management among adolescents. Effort should be made to combat this problem with a multipronged approach such as nutritional supplementation, growth monitoring, health education, encouraging physical activities in schools, and nutritional education. In addition, parents should be educated on these important behaviours.

## Figures and Tables

**Table 1.** Distribution of study participants according to age and gender

Age (yr)	Gender		Total
	Male	Female	
12	146 (51.6)	137 (48.4)	283 (34.9)
13	121 (62.7)	72 (37.3)	193 (23.8)
14	105 (61.8)	65 (38.2)	170 (21.0)
15	87 (53.0)	77 (47.0)	164 (20.2)
Total	459 (56.7)	351 (43.3)	810 (100)

Values are presented as number (%).

Chi-square value=8.499; p=0.037.

**Table 2.** Distribution of body mass index by gender, age, socioeconomic status (SES), school type, and diet

	Underweight (n=110)	Normal (n=473)	Overweight (n=184)	Obesity (n=43)	Total	p-value
Gender						
Male	74 (16.1)	251 (54.7)	95 (20.7)	39 (8.5)	459 (56.7)	<0.001
Female	36 (10.3)	222 (63.2)	89 (25.4)	4 (1.1)	351 (43.3)	
Age (yr)						
12	30 (27.3)	168 (35.5)	70 (38.0)	15 (34.9)	283 (34.9)	0.04
13	33 (30.0)	107 (22.6)	45 (24.5)	8 (18.6)	103 (23.8)	
14	21 (19.1)	112 (23.7)	33 (17.9)	4 (9.3)	170 (21.0)	
15	26 (23.6)	86 (18.2)	36 (19.6)	16 (37.2)	164 (20.2)	
SES^1^						
Upper high	16 (14.5)	37 (7.8)	30 (16.3)	5 (11.6)	88 (10.9)	<0.001
High	8 (7.3)	123 (26.0)	64 (34.8)	16 (37.2)	211 (26.0)	
Upper middle	42 (38.2)	161 (34.0)	47 (25.5)	16 (37.2)	266 (32.8)	
Lower middle	41 (37.3)	132 (27.9)	40 (21.7)	5 (11.6)	218 (26.9)	
Poor	3 (2.7)	20 (4.2)	3 (1.6)	1 (2.3)	27 (3.3)	
School type						
Private	33 (10.4)	165 (52.2)	99 (31.4)	19 (6.0)	316 (39.0)	<0.001
Government	77 (15.6)	308 (62.3)	85 (17.2)	24 (4.9)	494 (61.0)	
Diet						
Vegetarian	94 (13.5)	405 (57.6)	160 (22.9)	42 (6.0)	698 (86.2)	0.09
Mixed	16 (14.3)	71 (63.4)	24 (21.4)	1 (0.9)	112 (13.8)	
Total	110 (13.6)	473 (58.4)	184 (22.7)	43 (5.3)	810 (100)	

Values are presented as number (%).

1 SES was categorized based on Prasad’s socioeconomic classification.

**Table 3.** Mean daily diet score according to body mass index

Score	Underweight (n=110)	Normal (n=473)	Overweight (n=184)	Obese (n=43)	p-value
Food group	24.67±2.23	27.15±3.90	29.81±4.26	28.88±3.44	0.001
Nutrient	40.86±4.70	44.64±4.22	47.48±4.50	45.30±3.96	0.001
Sweet	10.34±3.96	10.45±4.72	15.01±6.56	15.76±7.29	0.001
Oral health diet	55±7.86	60.57±9.60	62.29±6.38	58.41±7.47	0.001

Values are presented as mean±SD.

**Table 4.** Relationship between body mass index and covariates using a multinomial regression

Variables		Underweight (n=110)	Overweight (n=184)	Obese (n=43)
Gender	Male	1.00	1.00	7.54 (2.63-21.61)^*^
	Female	1.72 (1.09-2.71)^*^	0.87 (0.61-1.25)	1.00
Age (yr)	12	0.69 (0.37-1.25)	1.13 (0.69-1.85)	0.47 (0.22-1.03)^*^
	13	1.01 (0.55-1.85)	1.12 (0.65-1.91)	0.38 (0.15-0.96)
	14	0.60 (0.31-1.16)	0.71 (0.40-1.25)	0.17 (0.05-0.55)^*^
	15	1.00	1.00	1.00
Socioeconomic status (SES)^1^	Upper high	1.18 (0.26-5.33)	3.53 (0.84-14.93)^*^	7.09 (0.60-83.49)
	High	0.18 (0.03-0.90)^*^	2.02 (0.49-8.32)	9.15 (0.77-108.11)^*^
	Upper Middle	1.81 (0.51-6.47)	2.12 (0.60-7.49)	2.35 (0.29-18.91)
	Lower Middle	2.22 (0.62-7.92)	2.01 (0.57-7.16)	0.77 (0.08-7.02)
	Poor	1.00	1.00	1.00
School type	Government	2.62 (1.20-5.71)^*^	2.08 (1.01-4.29)^*^	0.38 (0.09-1.56)
	Private	1.00	1.00	1.00
Diet	Vegetarian	0.73 (0.38-1.38)	1.46 (0.87-2.48)	10.74 (1.41-81.75)^*^
	Mixed	1.00	1.00	1.00

Values are presented as odds ratio (95% confidence interval).

1 SES was categorized based on Prasad’s socioeconomic classification.

* p<0.05.

## Appendix 1. Sample size calculation [7]

### Step 1: Base Sample-Size Calculation

The appropriate sample size for a population-based survey is determml:mined largely by three factors: (i) the estimated prevalence of the variable of interest, which is deviation from body mass index in this instance (the prevalence from our pilot study was calculated to be 50%), (ii) the desired level of confidence, and (iii) the acceptable margin of error.

Formula:

$$n\;=\frac{\;t²\times p(1-p)}{m²}$$

Description:

n=required sample sizet=confidence level at 95% (standard value of 1.96)p=estimated prevalence of variable of interest in the project aream=margin of error at 5% (standard value of 0.05)

Calculation:

$$n\;=\frac{1.96²\times 0.5\;(1-0.5)}{0.05²}$$

$$n\;=\frac{3.8416\times 0.25}{0.0025}$$

$$n\;=\frac{0.9604}{0.0025}$$

n =384.16-384

### Step 2: Design Effect

The anthropometric survey is designed as a cluster sample (a representative selection of villages), not a simple random sample. To correct for the difference in design, the sample size is multiplied by the design effect (D).

The design effect is generally assumed to be 2 for nutrition surveys using cluster-sampling methodology.

Calculation:

n×D=384×2=768

### Step 3: Contingency

The sample is further increased by 5% to account for contingencies such as non-response or recording errors.

Calculation:

n+5%=768×1.05=806.4-810
